# Loss of Deacetylation Enzymes Hdac6 and Sirt2 Promotes Acetylation of Cytoplasmic Tubulin, but Suppresses Axonemal Acetylation in Zebrafish Cilia

**DOI:** 10.3389/fcell.2021.676214

**Published:** 2021-06-28

**Authors:** Paweł K. Łysyganicz, Niedharsan Pooranachandran, Xinming Liu, Kathryn I. Adamson, Katarzyna Zielonka, Stone Elworthy, Fredericus J. van Eeden, Andrew J. Grierson, Jarema J. Malicki

**Affiliations:** ^1^The Bateson Centre, The University of Sheffield, Sheffield, United Kingdom; ^2^Department of Biomedical Science, University of Sheffield, Sheffield, United Kingdom; ^3^The School of Clinical Dentistry, The University of Sheffield, Sheffield, United Kingdom; ^4^Sheffield Institute for Translational Neuroscience, University of Sheffield, Sheffield, United Kingdom

**Keywords:** Hdac6, Sirt2, deacetylation, cilia, zebrafish

## Abstract

Cilia are evolutionarily highly conserved organelles with important functions in many organs. The extracellular component of the cilium protruding from the plasma membrane comprises an axoneme composed of microtubule doublets, arranged in a 9 + 0 conformation in primary cilia or 9 + 2 in motile cilia. These microtubules facilitate transport of intraflagellar cargoes along the axoneme. They also provide structural stability to the cilium, which may play an important role in sensory cilia, where signals are received from the movement of extracellular fluid. Post-translational modification of microtubules in cilia is a well-studied phenomenon, and acetylation on lysine 40 (K40) of alpha tubulin is prominent in cilia. It is believed that this modification contributes to the stabilization of cilia. Two classes of enzymes, histone acetyltransferases and histone deacetylases, mediate regulation of tubulin acetylation. Here we use a genetic approach, immunocytochemistry and behavioral tests to investigate the function of tubulin deacetylases in cilia in a zebrafish model. By mutating three histone deacetylase genes (*Sirt2*, *Hdac6*, and *Hdac10*), we identify an unforeseen role for Hdac6 and Sirt2 in cilia. As expected, mutation of these genes leads to increased acetylation of cytoplasmic tubulin, however, surprisingly it caused decreased tubulin acetylation in cilia in the developing eye, ear, brain and kidney. Cilia in the ear and eye showed elevated levels of mono-glycylated tubulin suggesting a compensatory mechanism. These changes did not affect the length or morphology of cilia, however, functional defects in balance was observed, suggesting that the level of tubulin acetylation may affect function of the cilium.

## Introduction

Cilia are microscopic microtubule-based structures necessary for the function of many signal transduction cascades, including those involved in vision, hearing, olfaction, and embryonic patterning (Omori and Malicki, 2006; Burcklé et al., 2011; Malicki, 2012). Cilia dysfunction is connected with the development of many diseases with a wide spectrum of phenotypes – including obesity, polydactyly, situs inversus, and retinal degeneration – collectively known as ciliopathies (Afzelius, 2004; Omori and Malicki, 2006; Omori et al., 2008; Waters and Beales, 2011; Borgal et al., 2012; Ran et al., 2015). Typically, a cilium contains nine doublets of outer microtubules (9 + 0). In addition, most motile cilia contain two doublets in the center (9 + 2) (Novarino et al., 2011). Microtubules are built from α and β tubulin subunits. However, a vast number of post-translational modifications, including de-tyrosination and the related Δ2 modifications, glutamylation, glycylation, palmitoylation, phosphorylation, and acetylation, affect not only dynamics of microtubules, but also their organization and interaction with other cellular components (Song and Brady, 2015). Acetylation is a dynamic and reversible process regulated by two classes of competing enzymes, histone acetyltransferases (HATs) and histone deacetylases (HDACs). Despite their nomenclature, these enzymes also act on many substrates other than histones, including tubulin. Acetylation of tubulin lysine 40 (K40) is believed to enhance microtubule and cilia stability and has been shown to affect cilia length in adipocytes (Nakakura et al., 2015; Forcioli-Conti et al., 2016). Furthermore, it has been suggested that acetylation of tubulin plays a role in kinesin-mediated transport (Reed et al., 2006). Importantly, deacetylation of tubulin in the axoneme was shown to be one of the events initiating cilia disassembly in adipocytes, hTERT-RPE1, IMCD-3 murine, and Caki-1 human renal cell lines, suggesting it has a critical role in ciliary dynamics (Pugacheva et al., 2007; Loktev et al., 2008; Forcioli-Conti et al., 2016).

HDACs are divided into 4 classes: class 1 (HDAC1, 2, 3, and 8), class 2a (HDAC4, 5, 7, and 9), class 2b (Hdac6 and 10), class 3 (SIRT1-7) and class 4 (HDAC11). The common features of classes 1, 2, and 4 are a zinc-dependent catalytic domain with a high degree of homology, and a number of accessory domains with regulatory functions (Miyake et al., 2016). In contrast, class 3 members are called sirtuins, which are NAD-dependent deacetylase enzymes and are related to the yeast protein Sir2 and evolved separately from other classes (Menegola et al., 2006; Guo et al., 2007; Zhao et al., 2010). Class 1 enzymes are widely expressed; class 2 and 4 enzymes are tissue-specific; class 3 is mostly expressed in neurons and muscles. Moreover, class 1 and 4 HDACs were reported to be nuclear, whereas class 2 and 3 HDACs shuttle between the nucleus and the cytoplasm (Menegola et al., 2006).

Hdac6 and Sirt2 were identified as the main cytoplasmic tubulin deacetylases (Hubbert et al., 2002; Inoue et al., 2007). Hdac6 was shown to localize in cilia and deacetylate axonemal tubulin which was suggested to be required for resorption of the cilium in various cell types (Pugacheva et al., 2007; Prodromou et al., 2012; Ran et al., 2015). This process was shown to be mediated by HEF-1 stabilized AurA1 kinase and Hdac6 phosphorylation (Pugacheva et al., 2007). Furthermore, interaction of Hdac6 with other proteins such as death inducer obliterator (Dido3) or cortactin was shown to affect cilia length and cilium resorption, respectively (Luxton and Gundersen, 2007; Zhang et al., 2007; de Diego et al., 2014; Ran et al., 2015). Hdac5 is another HDAC suggested to be responsible for tubulin deacetylation, however, its functions seem to be limited to peripheral nervous system (PNS) neurons and linked with responding to injury, whereas Hdac6 function seems to be more uniform (Chen and Rolls, 2012).

Hdac6 is the only HDAC family member that contains a fully duplicated class I/II HDAC-homology domain. This feature allows identification of Hdac6 orthologs in invertebrates, *Drosophila melanogaster* and *Caenorhabditis elegans*, and plants such as *Arabidopsis thaliana* (Boyault et al., 2007). The distinctive feature of the Sir2 family is the presence of a ∼200-amino-acid domain responsible for the NAD+-dependent deacetylase activity, which is well conserved in a wide variety of proteins from yeast to humans (Vaquero et al., 2006). Conservation of these domains across the species is a result of their interaction with many proteins, having role in a multitude of biological processes including transcription, cell signaling, inflammation, protein degradation, cell survival, angiogenesis, cell motility and cilia resorption (Hook et al., 2002; Matsuyama et al., 2002; Aoyagi and Archer, 2005; Kwon et al., 2007; Pugacheva et al., 2007; Tang et al., 2007; Zhang et al., 2007, 2008; Kaluza et al., 2011; Riolo et al., 2012; Li et al., 2013; Zhou et al., 2014). Interestingly, recent bioinformatics data points to a high level of promoter conservation among HDAC genes with the exception of HDAC5, 7, and 10 (Boltz et al., 2019).

Data obtained in mice show that Hdac6 deficient mice develop normally and are fertile regardless of increased acetylation of tubulin in most tissues (Zhang et al., 2008; Fukada et al., 2012). However, these mice display hyperactivity and lower levels of anxiety (Fukada et al., 2012). Similarly, Sirt2 mice mutants do not show developmental problems and activity of Sirt2 in hippocampus was linked to alternation in depressive behaviors (Bobrowska et al., 2012; Liu et al., 2015). Role of Hdac10 in vertebrate development was not described up to date, however, mutations in Hdac10 were linked to poor prognosis regarding several cancer types (Hai et al., 2017; Islam et al., 2017; Li et al., 2020; Yu et al., 2021).

In order to study the role of deacetylases in cilia formation and function, we created loss of function zebrafish mutants in three deacetylases: *hdac6*, *hdac10*, and *sirt2*. Hdac6 and Sirt2 are well described tubulin deacetylases and both were shown to affect cilia *in vitro* (Jing et al., 2007; Pugacheva et al., 2007; Zhou et al., 2014; Ran et al., 2015). The function of Hdac10 is poorly understood, but because of high similarity of the amino acid sequence with Hdac6, we predicted it may have a role in tubulin deacetylation. We present evidence that tubulin acetylation is regulated by Hdac6 and Sirt2 in zebrafish. Surprisingly, we also show that tubulin acetylation in a subset of cilia is reduced in the *hdac6* mutant, and that the effect is enhanced when *hdac6* and *sirt2* are both mutated. These data suggest the existence of organelle-specific regulation of tubulin acetylation in cilia. Finally, our data suggest functional relevance of these changes, as triple mutant larvae display impaired balance recovery after disorientating stimuli.

## Results

### Generation of *hdac6*, *hdac10*, and *sirt2* Loss of Function Alleles in Zebrafish

**FIGURE 1:**
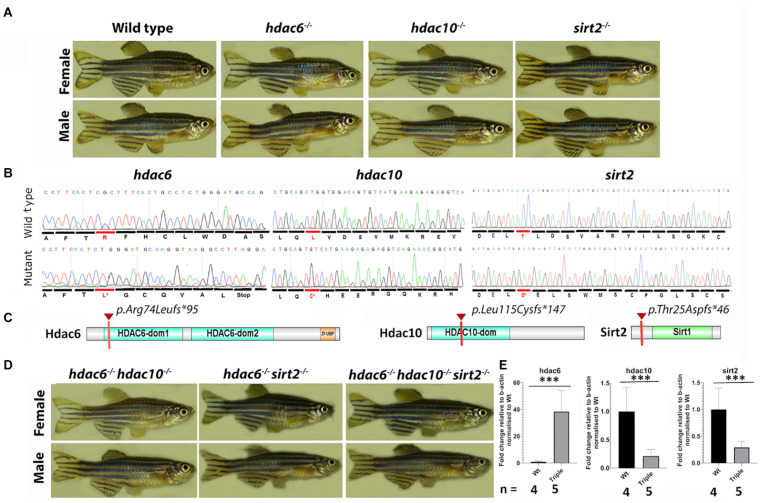
1.5-year-old zebrafish do not display any obvious external phenotype. **(A)** Pictures of single mutant fish were taken at 1.5 year – single mutation did not cause any visible abnormalities. **(B)** Comparison of wild type and mutated sequences for *hdac6* and *hdac10* and *sirt2* genes, the first changed amino acid caused by frameshift is indicated in red. **(C)** Schematic representation of proteins of interest – the red mark shows the premature stop codon caused by the mutation. **(D)** External phenotypes of double mutants and triple mutants. **(E)** qPCR results showing expression levels for hdac6, hdac10, and sirt2, relative to b-actin level and normalized to wild type (*t*-test ***p < 0.005).

To investigate the role of tubulin deacetylation in cilia assembly and maintenance, we generated mutants in two HDACs which contain Zn^2+^ binding domains: Hdac6 and the related Hdac10 (both class 2b), and in one NADH-dependent deacetylase: Sirt2. Mutations were generated using TALEN and CRISPR/Cas9 mutagenesis in zebrafish (*Danio rerio*) (Zu et al., 2013); Heler et al., 2014; Sander and Joung, 2014). By targeting *hdac6* with a TALEN, we obtained 3 mutant alleles containing deletions of 2, 8, and 14 bp. The 14 bp deletion allele (Figures 1A,B) was used for further analysis. This results in a frameshift and a premature stop codon at amino acid position 95 of 1081 (Figures 1B,C). Targeting the *hdac10* gene with CRISPR resulted in one mutant allele harboring an 11 bp deletion. This mutation also causes a frameshift and the appearance of a premature stop codon at amino acid position 147 of 676 (Figures 1B,C). Lastly, we used CRISPR to generate several *Sirt2* mutant alleles, including a 2 bp deletion, and a 2 bp deletion combined with a 24 bp insertion. The second allele, which results in a frameshift and a premature stop codon at amino acid position 46 of 379 (Figures 1B,C), was used in further studies. Although all three mutations are predicted to delete significant portions of the protein and conserved domains, homozygotes for these mutations do not display any obvious external phenotype, are viable, and fertile (Figure 1A). No overt defects were observed in homozygous progeny of homozygous in-crosses, excluding the possibility of maternal contribution of wild type mRNA masking any role in early development. To investigate whether these mutations lead to loss of function, we analyzed the levels of the relevant mRNA in 5 dpf larvae. This confirmed that *hdac*10 and *sirt*2 mutations lead to significant reductions in mRNA levels, indicating nonsense-mediated decay (Figure 1E and Supplementary Figure 1). However, the *hdac*6 mutation significantly elevated the level of its mRNA in single mutants, and this pattern was maintained in *hdac6; hdac10* and *hdac6; sirt2* double mutants, and *hdac6; hdac10; sirt2* triple mutant (hereafter named the Triple mutant) (Figure 1E and Supplementary Figure 1). Hdac6 might therefore negatively regulate its own expression at the transcriptional level. We did not detect cross-compensatory effects between these genes at the expression level.

Since Hdac6 and Hdac10 possess highly similar catalytic domains (although, the second domain of Hdac10 is described as inactive (de Ruijter et al., 2003; Tong et al., 2002), we hypothesized that they may act redundantly. To test this, we generated *hdac6;hdac10* double mutants. Similar to single mutants, these animals are viable and fertile and do not exhibit an overt external phenotype (Figure 1D). Since Hdac6 and Sirt2 were found to interact physically and catalyze tubulin deacetylation *in vitro* (North et al., 2003; Nahhas et al., 2007), we also generated *hdac6;sirt2* double mutants. Again, no overt phenotypes were visible, and double mutant fish were viable and fertile (Figure 1D). We showed that this was also the case for *hdac6; hdac10; sirt2* triple mutants (Figure 1D and Supplementary Figure 1). Therefore, the lack of a strong visible phenotype in single, double or triple mutants is not due to functional compensation involving these genes.

### Characterization of Tubulin Acetylation in *hdac6*, *hdac10*, and *sirt2* Mutant Zebrafish

**FIGURE 2:**
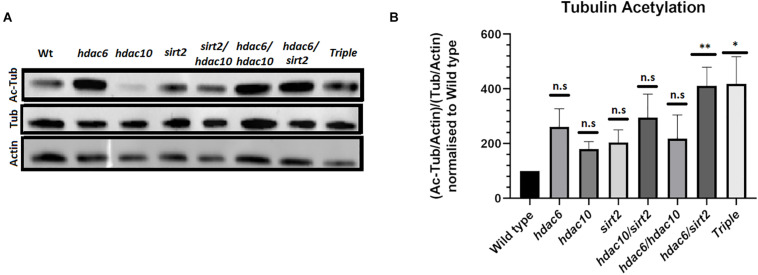
Level of tubulin acetylation in adult fish eyes. **(A)** Western blot showing changes in tubulin acetylation in *hdac* mutants. **(B)** Quantification of Western blot signals normalized to actin loading controls (Mean with SEM *n* = 4. One-way ANOVA *p < 0.05, **p < 0.01). ns, no significant.

**FIGURE 3:**
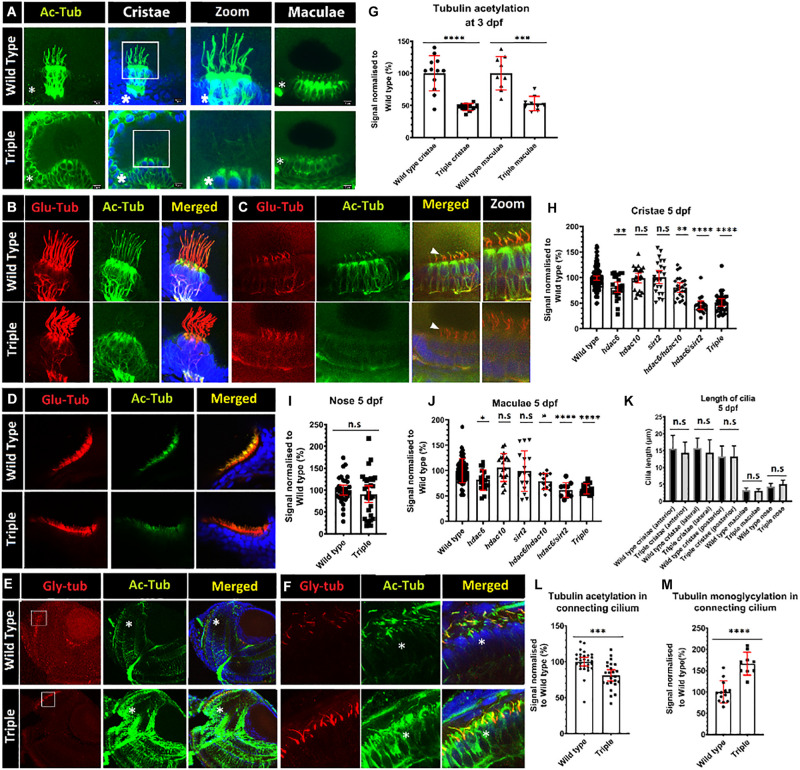
Decrease of acetylation in cilia in *hdac* mutants. **(A)** Comparison of levels of tubulin acetylation (green) between 3 dpf wild-type embryos and triple mutant embryos in cristae and macule. Squares shows zoomed in areas. **(B–D)** Double staining for acetylated (green) and glutamylated (red) tubulin, 5 dpf of wild type and triple mutants. **(B)** cristae **(C)** macule, and **(D)** nose. **(E)** Increased tubulin acetylation (green) of cell bodies in the retina of 5 dpf embryos, and increased levels of mono-glycylation in a subset of cilia (red). **(F)** Zoom of connecting cilia increased mono-glycylation as well as a lower level of acetylation in the triple mutants. **(G)** Level of tubulin acetylation in cilia in wild type and triple mutants, normalized to wild type, for cristae and macule of 3 dpf embryos. **(I)** Unaffected level of acetylation in nose cilia. Change of cristae **(H)** macule **(J)** and cilia tubulin acetylation in *hdac6, hdac10, sirt2, hdac6/hdac10, hdac6/sirt2*, and *triple* mutants. **(K)** Comparison of cilia length between wild type and triple mutants in cristae (anterior and lateral posterior) macule and nose. **(L)** Decrease in connecting cilium axoneme acetylation in photoreceptors in triple mutants and M) increase in tubulin mono-glycylation in triple mutant cilia. Asterisks show increased level of tubulin acetylation in cell bodies. [Mean with 95% CI. **(G,I,L,M)** *t*-test *p < 0.05, **p < 0.01, ***p < 0.005, ****p < 0.0001]. ns, no significant. [**(H,J,K)** one-way ANOVA *p < 0.05, **p < 0.01, ***p < 0.005, ****p < 0.0001]. ns, no significant.

To investigate whether loss of function alleles in *hdac6*, *hdac10*, and *sirt2* affect levels of tubulin acetylation *in vivo* we examined protein extracts from the eyes of single, double and triple mutant adults by Western Blotting. Each mutation appears to result in a higher level of tubulin acetylation when normalized to overall tubulin level (Figures 2A,B). The effect is enhanced and becomes statistically significant in the double mutants of *hdac6* and *sirt2*. The mutation in *hdac10* has only minimal or no effect on tubulin acetylation. Tubulin acetylation in triple mutant eye tissue does not differ from double mutants for *hdac6* and *sirt2* (Figures 2A,B). This finding is further supported by immunostaining of zebrafish eye cryosections (Figures 3E,F) and immunostaining of wholemount embryos showing elevated levels of acetylated tubulin within embryonic tissues (Supplementary Figure 2).

### Acetylation of Ciliary Axonemal Tubulin

**FIGURE 4:**
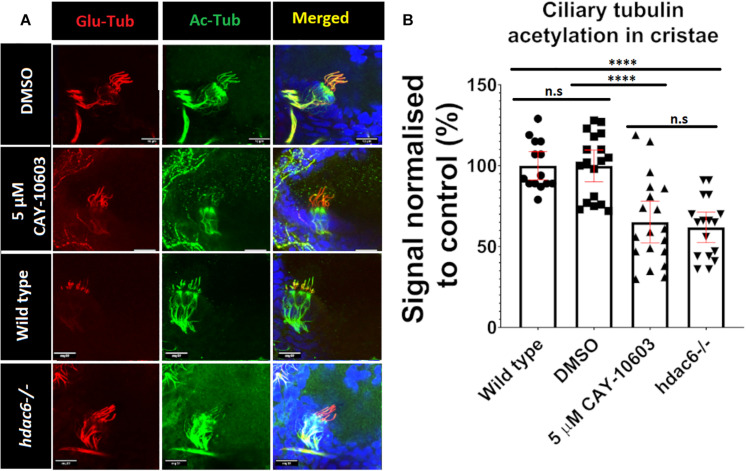
Chemical inhibition of Hdac6. **(A)** Effects of 72-h treatment with 5 μM CAY-10603 hdac6 inhibitor and *hdac6-/-* mutation on tubulin acetylation in cristae cilia of 3 dpf old embryo. **(B)** Tubulin acetylation level normalized to wt. Arrowheads point cilia. (*t*-test ****p < 0.0001. ns, no significant. *n*_*wt*_ = 32 *n*_*hdac*6_ = 17 *n*_*CAY*10603_ = 19).

Tubulin acetylation is associated with long lived microtubules such as axonemal microtubules (Janke and Montagnac, 2017). We reasoned that disrupting tubulin deacetylating enzymes may affect cilia morphology and function, as inhibition of Hdac6 was shown to stabilize cilia from regulated resorption (Luxton and Gundersen, 2007; Pugacheva et al., 2007; de Diego et al., 2014; Ran et al., 2015). To evaluate acetylation of ciliary microtubules, we immunostained triple mutant embryos using anti-acetyl-α-tubulin (Lys40) (D20G3), a well-characterized and highly specific antibody that recognizes acetylated tubulin and is commonly used in cilia visualization (Skoge and Ziegler, 2016; Viau et al., 2018; Sheu et al., 2019; Zhang et al., 2019). This analysis revealed increased acetylation of cytoplasmic microtubules in triple mutants compared to wild type (Figure 3A and Supplementary Figure 2). Surprisingly, we observed that hair cell kinocilia in cristae and macule of the inner ear in triple mutants are barely immunoreactive at 3 days post fertilization (dpf) (Figures 3A,G). This phenotype is observed as early as 1 dpf, when macule cilia are formed, and is also visible at 5 dpf (Figures 3B,C and Supplementary Figure 3). This phenotype is not limited to cilia in the ear and was observed in kidney, skin, and brain, at 1 dpf (Supplementary Figure 3). To evaluate this phenotype further, we investigated the impact of single and double knockouts on tubulin acetylation in cilia in the ear. *Hdac6* mutation decreased tubulin acetylation in kinocilia of cristae hair cells at 5 dpf by 20%, whereas mutations in either *hdac10* or *sirt2* did not show any significant effect. The combined mutation of *hdac6* and *sirt2* resulted in a decrease of ciliary tubulin acetylation by 60% compared to the control. *Hdac10* mutation did not affect ciliary tubulin acetylation even when combined with *hdac6*, or *hdac6*, and *sirt2* (Figure 3H). As reliable antibodies against zebrafish hdac6 are not commercially available to confirm the role of Hdac6 in the observed phenotype we utilized a chemical Hdac6 inhibitor, CAY-10603 (Figure 4 and Supplementary Figure 3) (Bitler et al., 2017). This drug was chosen over a more commonly used broad-range HDAC inhibitor trichostatin A (TSA) to avoid interference by inhibition of HDAC1, which severely affects zebrafish eye and inner ear development (Harrison et al., 2011; Finnin et al., 1999; Yamaguchi, 2005; He et al., 2016). We observed a tubulin hypoacetylation phenotype in kinocilia of cristae of wild-type zebrafish embryos treated with 5 μM of CAY-10603 from 6 to 72 hpf. Tubulin acetylation in cilia was decreased by 35% and was not significantly different from the *hdac6* single mutant (Figure 4). Only treatment at 6 h post fertilization resulted in the hypoacetylated phenotype in crista kinocilia, and fish treated at 24 and 48 hpf do not display this phenotype (Supplementary Figure 3). This is interesting as cilia in the ear are formed around 18 hpf, which would suggest that the observed hypoacetylation of axoneme occurs during ciliogenesis rather than being the result of later modifications (Whitfield, 2020).

To determine whether this phenotype is consistently observed in cilia of other tissues, we investigated kinocilia in inner ear macule, the connecting cilium of photoreceptor cells in the eye, and cilia in the nasal duct Tubulin acetylation in the kinocilia of inner ear macule was reduced in *hdac6, hdac10, sirt2* single mutants, and *hdac6; hdac10* double mutants. In the *hdac6; sirt2* double mutant and the triple mutant tubulin acetylation in cilia was reduced by 40%, compared with the control (Figure 3J). Tubulin acetylation in connecting cilia of photoreceptor cells of triple mutant retinae showed a 20% reduction compared to control (Figures 3E,F,L). Together these analyses show a consistent effect of loss of function of *hdac6* and *sirt2* on cilia tubulin acetylation *in vivo*. However, it appears that the magnitude of this effect differs in different cells, being strongest in cristae, intermediate in macule, and weakest in the specialized connecting cilium found in photoreceptors, whereas acetylation of cytoplasmic tubulin is uniformly increased (Figures 3A,E and Supplementary Figure 2). Finally, olfactory cilia in the nose of triple mutant embryos displayed normal levels of tubulin acetylation in cilia (Figures 3D,I).

**FIGURE 5:**
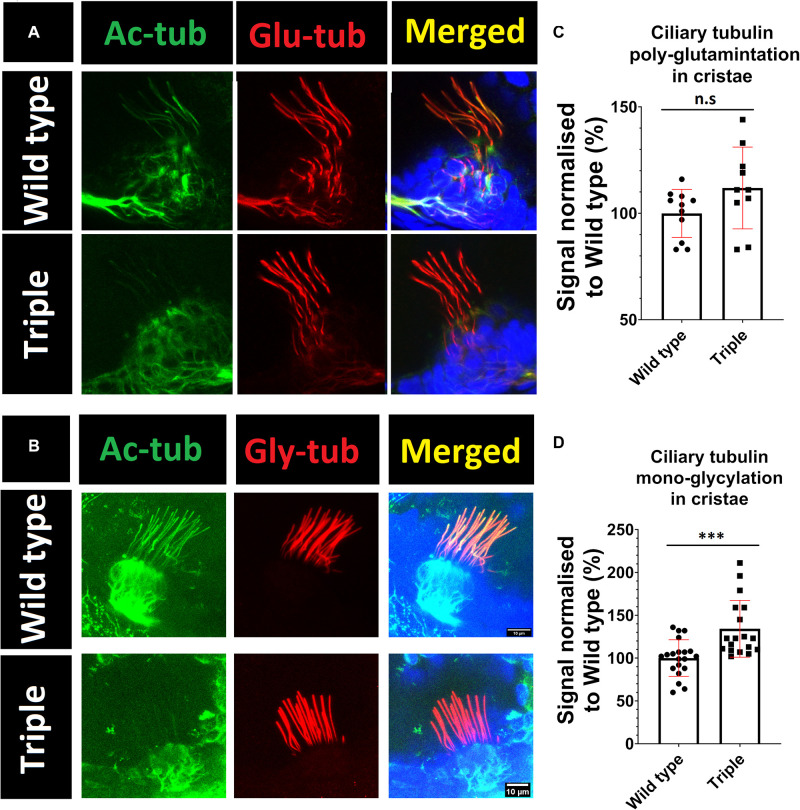
Increase in mono-glycylation, but not poly-glutamination, of tubulin in triple mutant cilia. Green acetylated tubulin, red **(A)** poly-glutamylated tubulin **(B)** mono-glycylated tubulin Blue DAPI. **(C,D)** signals normalized to wild type. *t*-test ***p < 0.05. ns, no significant.

To investigate possible abnormalities in cilia morphology, and the potential impact on other tubulin modifications that may modulate ciliary stability, embryos were stained with the following antibodies: GT335, against poly-glutamylated tubulin, and TAP952, against mono-glycylated tubulin. These experiments showed that *hdac6*, *sirt2*, or *hdac10* knockout does not affect ciliary axoneme length or shape in cristae or macule (Figure 3K). Our analysis indicates that lower levels of tubulin acetylation in the axoneme of triple mutants is correlated with increased levels of tubulin mono-glycylation, but not tubulin glutamylation, in ear kinocilia (Figures 5A–D) and in the connecting cilium of photoreceptor cells (Figures 3E,F,M).

**FIGURE 6:**
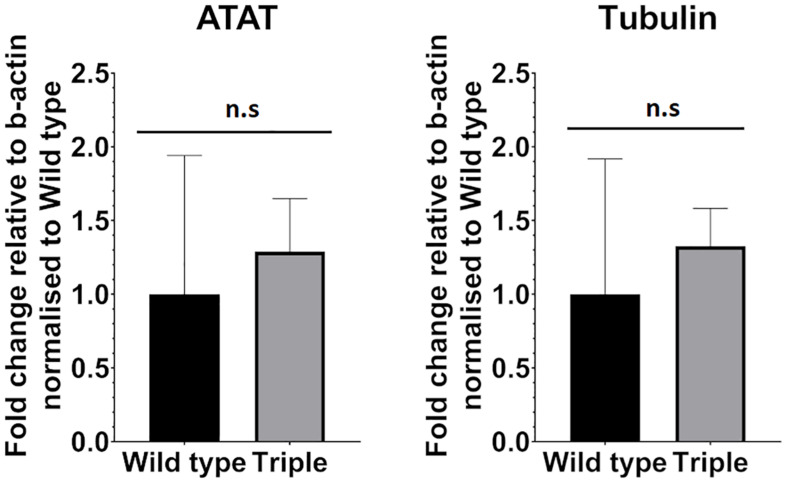
ATAT-1 and Tubulin mRNA expression levels relative to b-actin expression in Wild type and triple mutants. (*n*_wt_ = 4 *n*_tri_ = 6 mean with 95% CI.). ns, no significant.

We also considered the possibility that increased tubulin acetylation leads to reduced expression of *alpha-tubulin acetyltransferase1* gene (ATAT-1). A lower level of acetylated ciliary tubulin, we hypothesized, could be a response to elevated acetylation levels inside the cell body leading to decreased expression levels of ATAT-1 via negative feedback. To test this, we compared expression levels between wild-type and triple mutant embryos in 5 dpf larvae, using qPCR. This analysis shows that the level of ATAT-1 expression is not changed significantly in response to mutation in *hdac6, hdac10*, and *sirt2* genes (Figure 6).

### Loss of HDAC Genes Leads to Behavioral Changes

**FIGURE 7:**
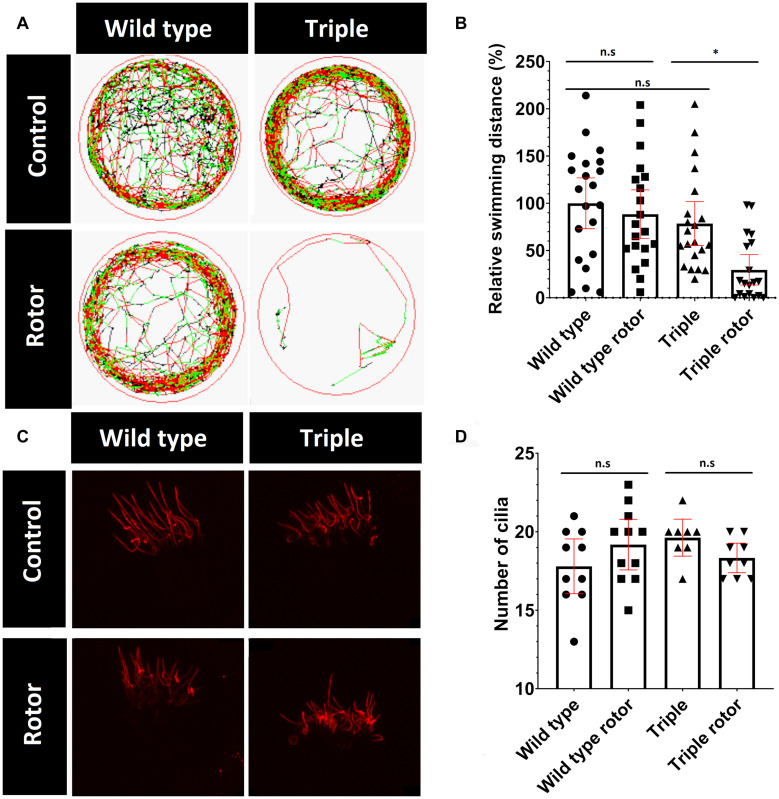
Change in mutant behavior. **(A)** Tracks of swimming patterns of Wild type and Triple mutant fish treated and untreated with disorientating stimulus. Black lines correspond to slow swimming (<5 mm/s), green lines indicate medium speed (5–10 mm/s) and red lines indicate fast swimming (>10 mm/s). The figure shows a representative swimming pattern of one larvae. **(B)** Swimming distance normalized to swimming distance by non-treated wild types. **(C)** TAP952 stained cilia in cristae of treated and untreated fish. **(D)** Number of cilia in cristae (mean with 95% CI. *T*-test *p < 0.05). ns, no significant.

Kinocilia in the inner-ear are responsible for detecting both static and dynamic balance. Static balance is the response to linear motion and gravity, which is detected by the utricle and saccule located within the vestibule (Forcioli-Conti et al., 2016). Dynamic balance involves the response to rotational motion such as turning. This is detected by cristae cilia in the semi-circular canals in response to movement of the gel-like cupula, in which the kinocilia are embedded and are fully functional as early as 3 weeks of age (Akella et al., 2010; Baxendale and Whitfield, 2016). Cilia damage in the inner ear and ciliopathies such as Usher syndrome are shown to affect the vestibular system (Renga, 2019; Whatley et al., 2020). Growing evidence links acetylation of tubulin to rigidity and resistance to mechanical stress (Szyk et al., 2014; Portran et al., 2017, Eshun-Wilson et al., 2019). Therefore, we hypothesized that cilia containing hypoacetylated tubulin, in the triple mutants, may be more vulnerable to accumulative mechanical damage. To evaluate this hypothesis, we induced mechanical stress on cilia in the ear, by placing tubes containing 5 dpf larvae (in E3 solution) on a vertical rotor at 30 rpm, for 30 min. Similar to the effect of a roller coaster in humans, movement of the otolith, physically coupled to hair cell kinocilia in the macule, should exert force, due to gravity and centrifugal forces, which disorientates the larvae. To quantify the ability of zebrafish larvae to recover from mechanical stress, we measured their recovery immediately after being removed from the rotor and placed in a 10cm dish. After 30 min of rotation, wild type fish commenced swimming within 1 min, whereas the majority of the triple mutant fish remained immotile for 20 min (Figure 7 and Supplementary Figure 4). Without introduction of stimulus, wild type and triple mutant swimming patterns did not visibly differ (Figure 7). These data show that the triple mutant was significantly more susceptible to balance loss after stimulus. To investigate possible cilia damage, we visualized ciliary tubulin using the anti-mono-glycylated tubulin antibody (TAP952) (Figure 7). The staining did not reveal any visible damage or breaks in ciliary axonemes. Furthermore, no difference in the number of cilia was observed (Figure 7).

## Discussion

In keeping with existing mouse models (Fukada et al., 2012), we find that inactivation of *hdac6* or *sirt2* does not cause a visible external phenotype in *D. rerio.* No overt phenotypes characteristic of cilia dysfunction, such as body curvature, hydrocephalus, kidney cysts or retinal degeneration were observed in homozygous mutants or their progeny, ruling out maternal contribution as a modifying factor (Sullivan-Brown et al., 2008; Shi et al., 2017; Maerz et al., 2019).

The critical role of cilia in a variety of biological processes, from early embryonic development, signal transduction and metabolism, to its role in mechanical functions such as sperm motility or synchronized wave-like movements removing mucus from the lungs connects these organelles with a myriad of diseases, syndromes, and dysfunctions (Malicki and Johnson, 2017; Reiter and Leroux, 2017). Here we describe effects of hdac6 and sirt2 proteins on tubulin acetylation in cilia, in a subset of zebrafish tissues, including cilia in the retina, ear, kidney, and brain. *In vitro* data suggests a crucial role of Hdac6 in resorption of primary cilia, as deacetylation of axonemal tubulin was suggested to be the event triggering the process (Pugacheva et al., 2007; Ran et al., 2015). Therefore, our prediction was that *hdac6* mutants would have increased acetylation and perhaps defects in resorption as a result what would lead to prolonged detectability of non-tether cilia in zebrafish otic vesicle (Stooke-Vaughan et al., 2012). Additionally, Hdac6 interacts with other proteins contributing to cilia resorption such as DIDO3 and cortactin furthermore strengthening our predictions (Pinho et al., 2016). Moreover tubulin acetylation is linked to efficiency of kinesin transport along microtubules, and kinesin mutations can the affect the presence or size of cilia (Reed et al., 2006; Pooranachandran and Malicki, 2016; Balabanian et al., 2017; Novas et al., 2018). Contrary to our expectations, mutants did not show any difference in length, or number of cilia in cristae. This is in contrast with published findings that Sirt2 and Hdac6 control cilium length in adipocytes, and suggests that roles of these genes in cilia maintenance are cell type specific (Forcioli-Conti et al., 2016).

Western blot analysis indicated that the level of tubulin acetylation was elevated in *hdac6*, *hdac10* and *sirt2* mutants and their combinations, particularly in *hdac6* and *sirt2* double mutant. The deacetylase domains of Hdac6 and Hdac10 are very similar, suggesting that in hdac6;sirt2 double mutants there may be compensation by Hdac10. By mutating *hdac10* in addition to *hdac6* and *sirt2* we excluded any possible redundancy in tubulin acetylation. However, a recent *in vitro* study has shown low catalytic activity of Hdac10 toward acetylated tubulin and suggested that its primary substrate is N8-acetylspermidine (Hai et al., 2017). Various studies link spermidine homeostasis with increased acetylation of structures involved in retrograde transport of autophagosomes, including microtubules (Phadwal et al., 2018).

Surprisingly, and contrary to our expectations, mutation in *hdac6* caused tubulin hypoacetylation in ear kinocilia and this phenotype was enhanced when combined with a mutation in *sirt2*. This phenotype was also observed in motile cilia in the brain and kidney as well as in macule kinocilia in larvae at 1 dpf. These observations may imply that either transport of the acetylase responsible for tubulin acetylation into the cilium is impaired, or that the predominant tubulin acetylase enzyme is regulated by hdac6 and sirt2. A similar but less pronounced phenotype was observed in connecting cilia of photoreceptors in the retina. However, tubulin acetylation in cilia in the olfactory system was not affected. This suggests tissue specificity of the phenomenon. Extensive research by Nakakura describing ATAT-1 localization in various rat tissues (Nakakura et al., 2016), revealed that the mechanisms by which ATAT-1 acetylates microtubules differ between tissues. ATAT-1 not only localizes to different intracellular compartments of different tissues, but sometimes do not correspond with acetylated microtubules. This difference in mechanism of acetylation may explain the differences observed in our study between the cilia of the olfactory system and other tissues.

We observed the same pattern in the cristae cilia acetylation using chemical inhibition of Hdac6, which also resulted in reduced ciliary tubulin acetylation when treatment was initiated at 6 hpf. A possible explanation for this observation is that cells react to high tubulin acetylation by reducing expression of *atat-1*, the enzyme that acetylates K40 tubulin in cilia. However, our experiments show that this does not occur at the transcriptional level, as there is no difference in *atat-1* mRNA expression between wild type and triple mutants. A potential caveat here is that we analyzed mRNA from whole larva, rather than isolated ciliated cells. It has been reported that mice with a loss of function mutation in ATAT-1, which have reduced levels of acetylated tubulin in cilia (Kim et al., 2013), also had normal primary and motile cilia length and morphology in the oviduct and the hippocampus (Kalebic et al., 2013). This is consistent with our observations and questions the importance of tubulin acetylation in cilia *in vivo*. Normal levels of atat-1 mRNA expression suggest that the observed phenotype may be connected with transport into cilium, rather than existence of a feedback loop controlling tubulin acetylation. However, zebrafish depleted in atat-1 (previously known a mec-17) display a drastic decrease of tubulin acetylation in neurons but not cilia, raising the possibility that in zebrafish other proteins may be responsible for acetylation of ciliary microtubules (Akella et al., 2010).

It is possible that other post-translational modifications of tubulin in cilia can contribute to axoneme stabilization in absence of acetylation. Our observation of elevated levels of mono-glycylation makes glycylation a likely candidate. This hypothesis is supported by the literature as tubulin glycylation was shown to mark and stabilize cilia, and mutation of glycylases results in fewer and shorter cilia (Wloga et al., 2009; Rocha et al., 2014). Interesting, there were differences in cilia glycylation between different cell types in cultured media (Gadadhar et al., 2017). For example, IMCD3 and RPE-1 cilia were shown not to be glycylated due to their short length, whereas, long cilia of MDCK were glycylated (Gadadhar et al., 2017). This strengthens the possibility that glycylation of axoneme plays an important role in stabilization of at least long cilia. Furthermore, unaffected levels of tubulin glutamylation it triple mutants strengthen this idea as it was previously shown that loss of cilia specific glutamylases resulted in flagella shortening in *Chlamydomonas*, and shortening of the 7th doublet of microtubules in mouse spermatozoa (Kubo et al., 2014; Konno et al., 2016).

Ciliopathies such as Usher syndrome and cilia damage are reported to affect vestibular system of patients and elderly, here we present data showing that mutation of both *hdac*6 and *sirt2* leads to impaired recovery from balance disruption after stimulation (Burns and Stone, 2017; Whatley et al., 2020). It may be associated with the speculated increase in axoneme fragility of cilia, as non-acetylated microtubules were proven to be more susceptible to mechanical stress (Szyk et al., 2014; Portran et al., 2017, Eshun-Wilson et al., 2019). However, staining with TAP952 antibodies did not reveal any clear damage to the ciliary axoneme. A possible explanation for this observation is that non-acetylated microtubules were shown to bend more than their acetylated counterparts. It is possible that changes in rigidity of cilia in cristae and macule result in misinterpretation of the stimuli. Alternatively, the slow recovery may be attributed to effects of hdac6/sirt2 mutation on tubulin in the nervous system, since behavioral changes have been reported in both zebrafish and mice (Fukada et al., 2012; Pinho et al., 2016).

In this study, we show that acetylation is not necessary for the maintenance of stable cilia *in vivo*, and contrary to previous beliefs, loss of deacetylases did not enhance but rather reduced ciliary microtubule acetylation. Interestingly, zebrafish with hypoacetylated cilia display defective balance recovery, however, whether this is correlated with the acetylation status of the axoneme, or other functions of hdac6, will need further evaluation.

## Materials and Methods

### Zebrafish Strains and Maintenance

Hdac6 alleles were obtained using TALEN (Zu et al., 2013) (target site TTCACTCGCTTTCACTGCC TALEN binding sequences: GTATATGTAGACGCC and TCTGGGATGCCAGGT deleted 14 bp GCTTTCACTGCCTC name SH400). hdac10 (target site GGGAGCAACTCTGCAGCTGG Oligo1:TAGGGAGCAA CTCTGCAGCTGG Oligo2: AAACCCAGCTGCAGAGTTGCTC deleted 11 bp CTGGTGGACAG name SH401) and sirt2 (target site AGTCTTGGATGAGTTAACCC Oligo1: CACTT GGCCTTAGTCCTGGA Oligo2 GCACAGCAGCAAAGTGT TCA deleted 2 bp AA then inserted 24 bp GGATGAGT TGTCTTGGATGAGTTG name SH626) were created with CRISPR/Cas9 (Heler et al., 2014; Sander and Joung, 2014). Double and triple mutants were obtained by crossing parental strains and identified by DNA sequencing. Zebrafish were maintained in accordance with the UK Home Office regulations and UK Animals (Scientific Procedures Act 1986).

### Immunohistochemistry and Microscopy

Sectioning and immunohistochemistry were performed using previously described protocols (Malicki et al., 2011). Following fixation, transverse cryosections, 20 μm thick, were taken through the retina or the ear and processed for staining with the following antibodies: anti-acetylated a-tubulin (rabbit) (D20G3; 1:1000; Cell signaling) Acetyl-α-Tubulin (Lys40) (mouse) (6-11B-1), anti-polyglutaminated tubulin GT335 (1:500; Adipogen) anti-monoglycylated tubulin TAP952 (1:500; Millipore) (Alexa Fluor 488- and 564-conjugated secondary antibodies were used for all staining procedures (1:1000) as well as DAPI for counterstaining. Images of cryosections were collected at the Wolfson Light Microscopy Facility using an Olympus FV1000 confocal microscope and a 60x immersion objective. Images of immunostained wholemount embryos were collected using the Olympus FV1000 confocal microscope with a 40× dipping lens.

### Western Blotting

Eyes of 2.5-year-old fish were dissected and subsequently snap frozen in liquid nitrogen. Eyes were homogenized in RIPA buffer (Catalog No.: 89900 Thermo Fisher Scientific) and centrifuged supernatant was transferred to Eppendorf tubes and used for the Western Blotting. Proteins were resolved by SDS–PAGE, transferred to nitrocellulose membrane (Amersham GEHealthcare), and incubated with antibodies C4 (MAB1501R Sigma-Aldrich) for actin and α-Tubulin (DM1A) (Mouse mAb #3873) for tubulin and Acetyl-α-Tubulin (Lys40) (6-11B-1) for acetylated tubulin. For detection an Amersham ECL Western Blotting Detection Kit was used as described in manual. To visualize blots a ChemiDoc MP Imaging System was used and signals were measured using ImageJ.

### Quantitative Reverse Transcription-PCR Expression Analyses

RNA was extracted from ten 5 dpf embryos using the Monarch Total RNA Miniprep Kit (New England Biolabs Inc., United Kingdom) following the manufacturer’s instructions. Extracted RNA was quantified with a NanoDrop 1000 spectrophotometer (Thermo Fisher Scientific, United States). Reverse transcription of cDNA was performed using a High-Capacity cDNA Reverse Transcription Kit (Catalog No.: 4368814, Applied Biosystems, United Kingdom) following the manufacturer’s instructions. cDNA was kept at 4°C for immediate use or −20°C for long-time storage. cDNA was kept at 4°C for immediate use or −20°C for long term storage. qPCR reactions were carried out to quantify RNA expression of genes of interest using SYBR Green. Beta-actin was used as a housekeeping reference gene control.

Primers used: *hdac10* F5′-TGTCCTATGAAGCGCTCAGG-3′ R5′-TTTGACCGCCTCCAGGTACT-3′, *hdac6* F5′-GGAGATG AGCAATGAACTGCAA-3′ R5′-GACTGGCATCCCAGAGTGA A-3′, *sirt2* F5′-TTTGCGCAGTCTTTTCTCGC-3′ R5′-CCCAG CTCCAACCATACAGA-3′, *atat-1* F5′-TCCCTTACGACCTG AATGCG-3′ R5′-GATCTGGTCTCCCATGCGCT-3′, *tubulin* F5′-ATGCTCGTGGTCACTACACT-3′ R5′-GGAAACCCTGG AGACCTGTG-3′. Data Analysis for qPCR was conducted using Microsoft Excel 2010 (Microsoft Corporation, United States) and GraphPad Prism 8 (GraphPad Software, United States).

The threshold level of fluorescent intensity was set to 0.04 to obtain Ct values across all experiments. Average Ct values from three technical repeats were firstly calculated. ΔCt values were obtained by subtracting Ct values of beta-actin from Ct values of the target gene, from of the same biological sample. The fold change was calculated with the formula 2^–ΔCt^, and the standard deviation was automatically calculated and shown as error bars.

### Behavioral Tests

For balance tests, three 5 dpf zebrafish larvae were transferred to Eppendorf tubes and exposed to the centrifugal force of approximately 0.0005 N (*r* = 0.125 m *v* = 0.5 m/s *m* = 0.00025 g), using a Stuart^TM^ Rotator Disk for 30 min (40 rpm). Subsequently, fish were transferred to a 12-well plate and their behavior was recorded (Supplementary Figure 5). Swimming distances were normalized to Wild type.

### Chemical Treatment

Embryos were treated with 5 μM CAY10603 (cat. no: B2819-5, Cambridge Bioscience) for 1, 2, or 3 days, with the solution changed daily.

### Software and Data Aanalysis

Data from cryosections and whole-mount immunostaining were analyzed with Fiji (ImageJ) software. Schematic illustration of protein domains was created with biocuckoo software (Liu et al., 2015). Sequencing data was analyzed with SnapGene software.

## Data Availability Statement

The original contributions presented in the study are included in the article/Supplementary Material, further inquiries can be directed to the corresponding author/s.

## Ethics Statement

The animal study was reviewed and approved by UK Home Office regulations and UK Animals (Scientific Procedures Act 1986).

## Author Contributions

JM, PŁ, and AG: conceptualization. PŁ: data curation, formal analysis, behavioral tests, methodology behavioral test, and writing – original draft preparation. JM: funding acquisition and resources. NP, PŁ, and SE: investigation generation of mutants. PŁ, SE, and KZ: immunochemistry. PŁ and KZ: CAY-10603 inhibition. PŁ and KA: western blots. XL: qPCR. JM, PŁ, AG, and FE: project administration. JM, AG, and FE: supervision. JM, AG, FE, and PŁ: validation. PŁ, NP, AG, and FE: writing – review and editing. All authors contributed to the article and approved the submitted version.

## Conflict of Interest

The authors declare that the research was conducted in the absence of any commercial or financial relationships that could be construed as a potential conflict of interest.

## Dedication

This paper is dedicated to the memory of JM who sadly died while the work was in progress.

## Supplementary Material

Supplementary Figure 1 Levels of mRNA expression for all three genes in various single and double mutants. The mutant is indicated above the panel the gene that was analyzed for expression is indicated on the *X*-axis. Wt: pooled wild-type siblings. Mut: pooled mutants **(A–C)** or double mutants **(D,E) (A)** hdac6-/- **B)** hdac10-/- **(C)** sirt2-/- single mutants and **(D)** hdac6-/-/hdac10-/- and hdac6-/-/sirt2-/- double mutants. Values normalized to actin expression level and then to wild type values. (Mean with SEM for *n* = 3. *p* < 0.05).

Supplementary Figure 2 Hyperacetylation of tubulin in brain cells surrounding ear and kidney and hypoacetylation in cilia at 1 dpf. Red Acetyl-α-Tubulin (Lys40) (6-11B-1) Mouse mAb blue DAPI. Asterisks show hyperacetylated tubulin within cell bodies. Arrowheads shows cilia positions.

Supplementary Figure 3 Early inhibition (6 hpf) of hdac6 is required to trigger hypoacetylation phenotype in cilia in cristae at 3 dpf. Acetylated tubulin green glutamylated red DAPI blue. White boxes show cilia position. Mean with CI95% *n*_*wt*_ = 12 *t*_*reated*_ = 11. *p* < 0.05.

Supplementary Figure 4 Immotile fish after rotor treatment. Percent of fish which swam less than 20% of distance, swam by average of untreated wild type (three larvae per trail). Mean with CI-95% One-way ANOVA *p* < 0.05.

Supplementary Figure 5 Schematic representation of disturbing balance experiment in zebrafish larvae. Wt, Wild type; Wt r, Wild type after treatment; Tri, Triple mutant; Tri r, Triple mutant after treatment.

## References

[B1] Afzelius B. A. (2004). Cilia-related diseases. *J. Pathol*,. 204 470–477. 15495266 10.1002/path.1652 PMC7167937

[B2] Akella J. S. Wloga D. Kim J. Starostina N. G. Lyons-Abbott S. Morrissette N. S. (2010). MEC-17 is an α-tubulinacetyltransferase. *Nature* 467 218–222. 10.1038/nature0932 20829795 PMC2938957

[B3] Aoyagi S. Archer T. K. (2005). Modulating molecular chaperone Hsp90 functions through reversible acetylation. *Trends Cell Biol.* 15 565–567. 10.1016/j.tcb.2005.09.003 16199163

[B4] Balabanian L. Berger C. L. Hendricks A. G. (2017). Acetylated Microtubules Are Preferentially Bundled Leading to Enhanced Kinesin-1 Motility. *Biophys. J.* 113 1551–1560. 10.1016/j.bpj.2017.08.009 28978447 PMC5627185

[B5] Baxendale S. Whitfield T. T. (2016). Methods to study the development, anatomy, and function of the zebrafish inner ear across the life course. *Methods Cell Biol.* 134 165–209. 10.1016/bs.mcb.2016.02.007 27312494

[B6] Bitler B. G. Wu S. Park P. H. Hai Y. Aird K. M. Wang Y. (2017). ARID1A-mutated ovarian cancers depend on HDAC6 activity. *Nat. Cell Biol.* 19 962–973. 10.1038/ncb3582 28737768 PMC5541905

[B7] Bobrowska A. Donmez G. Weiss A. Guarente L. Bates G. (2012). SIRT2 ablation has no effect on tubulin acetylation in brain, cholesterol biosynthesis or the progression of huntington’s disease phenotypes *in vivo*. *PloS One* 7:e34805. 10.1371/journal.pone.0034805 22511966 PMC3325254

[B8] Boltz T. A. Khuri S. Wuchty S. (2019). Promoter conservation in HDACs points to functional implications. *BMC Genom.* 20:613. 10.1186/s12864-019-5973-x 31351464 PMC6660948

[B9] Borgal L. Habbig S. Hatzold J. Liebau M. C. Dafinger C. Sacarea I. (2012). The ciliary protein nephrocystin-4 translocates the canonical Wnt regulator Jade-1 to the nucleus to negatively regulate catenin signaling. *J. Biol. Chem.* 287 25370–25380. 10.1074/jbc.M112.385658 22654112 PMC3408186

[B10] Boyault C. Sadoul K. Pabion M. Khochbin S. (2007). HDAC6, at the crossroads between cytoskeleton and cell signaling by acetylation and ubiquitination. *Oncogene*, 26 5468–5476. 10.1038/sj.onc.1210614 17694087

[B11] Burcklé C. Gaudé H. M. Vesque C. Silbermann F. Salomon R. Jeanpierre C. (2011). Control of the Wnt pathways by nephrocystin-4 is required for morphogenesis of the zebrafish pronephros. *Hum. Mol. Genet.* 20 2611–2627. 10.1093/hmg/ddr164 21498478

[B12] Burns J. C. Stone J. S. (2017). Development and regeneration of vestibular hair cells in mammals. *Sem. Cell Dev. Biol.* 65 96–105. 10.1016/j.semcdb.2016.11.001 27864084 PMC5423856

[B13] Chen L. Rolls M. M. (2012). Microtubule deacetylation sets the stage for successful axon regeneration. *EMBO J.* 31 3033–3035. 10.1038/emboj.2012.175 22735188 PMC3400021

[B14] de Diego A. S. Alonso Guerrero A. Martínez-A C. van Wely K. H. M. (2014). Dido3-dependent HDAC6 targeting controls cilium size. *Nat. Commun.* 5 1–11. 10.1038/ncomms4500 24667272 PMC3973121

[B15] de Ruijter A. J. M. van Gennip A. H. Caron H. N. Kemp S. van Kuilenburg A. B. P. (2003). Histone deacetylases (HDACs): characterization of the classical HDAC family. *Biochem. J.* 370 737–749. 10.1042/BJ2002132 12429021 PMC1223209

[B16] Eshun-Wilson L. Zhang R. Portran D. Nachury M. V. Toso D. B. Löhr T. (2019). Effects of α-tubulin acetylation on microtubule structure and stability. *Proc. Nat. Acad. Sci. U. S. A.* 116 10366–10371. 10.1073/pnas.1900441116 31072936 PMC6535015

[B17] Finnin M. S. Donigian J. R. Cohen A. Richon V. M. Rifkind R. A. Marks P. A. (1999). Structures of a histone deacetylase homologue bound to the TSA and SAHA inhibitors. *Nature* 401 188–193. 10.1038/43710 10490031

[B18] Forcioli-Conti N. Estève D. Bouloumiè A. Dani C. Peraldi P. (2016). The size of the primary cilium and acetylated tubulin are modulated during adipocyte differentiation: analysis of HDAC6 functions in these processes. *Biochimie* 124 112–123. 10.1016/j.biochi.2015.09.011 26363102

[B19] Fukada M. Hanai A. Nakayama A. Suzuki T. Miyata N. Rodriguiz R. M. (2012). Loss of deacetylation activity of Hdac6 affects emotional behavior in mice. *PLoS One* 7:e30924. 10.1371/journal.pone.0030924 22328923 PMC3273475

[B20] Gadadhar S. Dadi H. Bodakuntla S. Schnitzler A. Bièche I. Rusconi F. (2017). Tubulin glycylation controls primary cilia length. *J. Cell Biol.* 216 2701–2713 10.1083/jcb.201612050 28687664 PMC5584158

[B21] Guo C. Mi J. Brautigan D. L. Larner J. M. (2007). ATM regulates ionizing radiation-induced disruption of HDAC1:PP1:Rb complexes. *Cell.lar Signal.* 19 504–510. 10.1016/j.cellsig.2006.08.001 17008050

[B22] Hai Y. Shin sky S. A. Porter N. J. Christianson D. W. (2017). Histone deacetylase 10 structure and molecular function as a polyamine deacetylase. *Nat. Commun.* 8:15368. 10.1038/ncomms15368 28516954 PMC5454378

[B23] Harrison M. R. M. Georgiou A. S. Spaink H. P. Cunliffe V. T. (2011). The epigenetic regulator Histone Deacetylase 1 promotes transcription of a core neurogenic programme in zebrafish embryos. *BMC Genom.* 12:24. 10.1186/1471-2164-12-24 21226904 PMC3032698

[B24] He Y. Tang D. Li W. Chai R. Li H. (2016). Histone deacetylase 1 is required for the development of the zebrafish inner ear. *Sci. Rep.* 6:16535. 10.1038/srep16535 26832938 PMC4735278

[B25] Heler R. Marraffini L. A. Bikard D. (2014). Adapting to new threats: the generation of memory by CRISPR-Cas immune systems. *Mol. Microbiol.* 93 1–9. 10.1111/mmi.12640 24806524 PMC4104294

[B26] Hook S. S. Orian A. Cowley S. M. Eisenman R. N. (2002). Histone deacetylase 6 binds polyubiquitin through its zinc finger (PAZ domain) and copurifies with deubiquitinating enzymes. *Proc. Natl. Acad. Sci.* 99, 13425–13430. 10.1073/pnas.172511699 12354939 PMC129689

[B27] Hubbert C. Guardiola A. Shao R. Kawaguchi Y. Ito A. Nixon A. (2002). HDAC6 is a microtubule-associated deacetylase. *Nature* 417 455–458. 10.1038/417455a 12024216

[B28] Inoue T. Hiratsuka M. Osaki M. Yamada H. Kishimoto I. Yamaguchi S. (2007). SIRT2, a tubulin deacetylase, acts to block the entry to chromosome condensation in response to mitotic stress. *Oncogene* 26 945–957. 10.1038/sj.onc.1209857 16909107

[B29] Islam M. M. Banerjee T. Packard C. Z. Kotian S. Selvendiran K. Cohn D. E. (2017). HDAC10 as a potential therapeutic target in ovarian cancer. *Gynecol. Oncol.* 144, 613–620. 10.1016/j.ygyno.2017.01.009 28073598 PMC6020686

[B30] Janke C. Montagnac G. (2017). Causes and Consequences of Microtubule Acetylation. *Curr. Biol.* 27 R1287–R1292. 10.1016/j.cub.2017.10.044 29207274

[B31] Jing E. Gesta S. Kahn C. R. (2007). SIRT2 Regulates Adipocyte Differentiation through FoxO1 Acetylation/Deacetylation. *Cell Metabol.* 6 105–114. 10.1016/j.cmet.2007.07.003 PMC2083635 17681146

[B32] Kalebic N. Sorrentino S. Perlas E. Bolasco G. Martinez C. Heppenstall P. A. (2013). αTAT1 is the major α-tubulin acetyltransferase in mice. *Nat. Commun.* 4:1962. 10.1038/ncomms2962 23748901

[B33] Kim G. W. Li L. Gorbani M. You L. Yang X. J. (2013). Mice lacking α-tubulin acetyltransferase 1 are viable but display α-tubulin acetylation deficiency and dentate gyrus distortion. *J. Biol. Chem.* 288, 20334–20350. 10.1074/jbc.M113.464792 23720746 PMC3711300

[B34] Kaluza D. Kroll J. Gesierich S. Yao T. P. Boon R. A. Hergenreider E. (2011). Class IIb HDAC6 regulates endothelial cell migration and angiogenesis by deacetylation of cortactin. *EMBO J.* 30 4142–4156. 10.1038/emboj.2011.298 21847094 PMC3199386

[B35] Konno A. Ikegami K. Konishi Y. Yang H. J. Abe M. Yamazaki M. (2016). Ttll9-/- mice sperm flagella show shortening of doublet 7, reduction of doublet 5 polyglutamylation and a stall in beating. *J. Cell Sci.* 129 2757–2766. 10.1242/jcs.185983 27257088

[B36] Kubo T. Yanagisawa H.-A. Liu Z. Shibuya R. Hirono M. Kamiya R. (2014). A conserved flagella-associated protein in Chlamydomonas, FAP234, is essential for axonemal localization of tubulin polyglutamylase TTLL9. *Mol. Biol. Cell* 25, 107–117. 10.1091/mbc.E13-07-0424 24196831 PMC3873882

[B37] Kwon S. Zhang Y. Matthias P. (2007). The deacetylase HDAC6 is an essential component of stress granules and plays a critical role in the cellular response to stress Inauguraldissertation. *Genes Dev.* 21 3381–3394. 10.1101/gad.461107.lates

[B38] Li Y. Shin D. Kwon S. H. (2013). Histone deacetylase 6 plays a role as a distinct regulator of diverse cellular processes. *FEBS J.* 280 775–793. 10.1111/febs.12079 23181831

[B39] Li Y. Zhang X. Zhu S. Dejene E. A. Peng W. Sepulveda A. (2020). HDAC10 Regulates Cancer Stem-Like Cell Properties in KRAS-Driven Lung Adenocarcinoma. *Cancer Res.* 80, 3265–3278. 10.1158/0008-5472.CAN-19-3613 32540961 PMC7442594

[B40] Liu R. Dang W. Du Y. Zhou Q. Jiao K. Liu Z. (2015). SIRT2 is involved in the modulation of depressive behaviors. *Sci. Rep.* 5:8415. 10.1038/srep08415 25672834 PMC4325337

[B41] Liu W. Xie Y. Ma J. Luo X. Nie P. Zuo Z. (2015). IBS: an illustrator for the presentation and visualization of biological sequences. *Bioinformatics* 31 3359–3361. 10.1093/bioinformatics/btv362 26069263 PMC4595897

[B42] Loktev A. V. Zhang Q. Beck J. S. Searby C. C. Scheetz T. E. Bazan J. F. (2008). A BBSome Subunit Links Ciliogenesis, Microtubule Stability, and Acetylation. *Dev. Cell* 15 854–865. 10.1016/j.devcel.2008.11.001 19081074

[B43] Luxton G. W. G. Gundersen G. G. (2007). HDAC6-Pack: cortactin Acetylation Joins the Brew. *Dev. Cell* 13 161–162. 10.1016/j.devcel.2007.07.014 17681125

[B44] Maerz L. D. Casar Tena T. Gerhards J. Donow C. Jeggo P. A. Philipp M. (2019). Analysis of cilia dysfunction phenotypes in zebrafish embryos depleted of Origin recognition complex factors. *Eur. J. Hum. Genet.* 27 772–782. 10.1038/s41431-019-0338-0 30696958 PMC6461852

[B45] Malicki J. (2012). Who drives the ciliary highway? *BioArchitecture* 2 111–117. 10.4161/bioa.21101 22960672 PMC3675070

[B46] Malicki J. Avanesov A. Li J. Yuan S. Sun Z. (2011). “Analysis of cilia structure and function in zebrafish,” in *Methods in Cell Biology*, 3rd Edn, (Elsevier Ltd), 101. 10.1016/B978-0-12-387036-0.00003-7 21550439

[B47] Malicki J. J. Johnson C. A. (2017). The Cilium: cellular Antenna and Central Processing Unit. *Trends Cell Biol.* 27 126–140. 10.1016/j.tcb.2016.08.002 27634431 PMC5278183

[B48] Matsuyama A. Shimazu T. Sumida Y. Saito A. Yoshimatsu Y. Seigneurin-Berny D. (2002). In vivo destabilization of dynamic microtubules by HDAC6-mediated deacetylation. *EMBO J.* 21 6820–6831. 10.1093/emboj/cdf682 12486003 PMC139102

[B49] Menegola E. Di Renzo F. Broccia M. L. Giavini E. (2006). Inhibition of histone deacetylase as a new mechanism of teratogenesis. *Birth Def. Res. Part C* 78 345–353. 10.1002/bdrc.20082 17315247

[B50] Miyake Y. Keusch J. J. Wang L. Saito M. Hess D. Wang X. (2016). Structural insights into HDAC6 tubulin deacetylation and its selective inhibition. *Nat. Chem. Biol.* 12 748–754. 10.1038/nchembio.2140 27454931

[B51] Nahhas F. Dryden S. C. Abrams J. Tainsky M. A. (2007). Mutations in SIRT2 deacetylase which regulate enzymatic activity but not its interaction with HDAC6 and tubulin. *Mol. Cell. Biochem.* 303 221–230. 10.1007/s11010-007-9478-6 17516032

[B52] Nakakura T. Asano-Hoshino A. Suzuki T. Arisawa K. Tanaka H. Sekino Y. (2015). The elongation of primary cilia via the acetylation of α-tubulin by the treatment with lithium chloride in human fibroblast KD cells. *Med. Mol. Morphol.* 48 44–53. 10.1007/s00795-014-0076-x 24760594

[B53] Nakakura T. Suzuki T. Nemoto T. Tanaka H. Asano-Hoshino A. Arisawa K. (2016). Intracellular localization of α-tubulin acetyltransferase ATAT1 in rat ciliated cells. *Med. Mol. Morphol.* 49 133–143. 10.1007/s00795-015-0132-1 26700226

[B54] North B. J. Marshall B. L. Borra M. T. Denu J. M. Verdin E. Francisco S. (2003). The Human Sir2 Ortholog, SIRT2, Is an NAD ^.^/.. -Dependent Tubulin Deacetylase. *Mol. Cell^∗^* 11 437–444. 10.1016/S1097-2765(3)00038-8 12620231

[B55] Novarino G. Akizu N. Gleeson J. G. (2011). Modeling human disease in humans: the ciliopathies. *Cell* 147 70–79. 10.1016/j.cell.2011.09.014 21962508 PMC3202432

[B56] Novas R. Cardenas-Rodriguez M. Lepanto P. Fabregat M. Rodao M. Fariello M. I. (2018). Kinesin 1 regulates cilia length through an interaction with the Bardet-Biedl syndrome related protein CCDC28B. *Sci. Rep.* 8 1–16. 10.1038/s41598-018-21329-6 29445114 PMC5813027

[B57] Omori Y. Malicki J. (2006). oko meduzy and Related crumbs Genes Are Determinants of Apical Cell Features in the Vertebrate Embryo. *Curr. Biol.* 16 945–957. 10.1016/j.cub.2006.03.058 16713951

[B58] Omori Y. Zhao C. Saras A. Mukhopadhyay S. Kim W. Furukawa T. (2008). Elipsa is an early determinant of ciliogenesis that links the IFT particle to membrane-associated small GTPase Rab8. *Nat. Cell Biol.* 10 437–444. 10.1038/ncb1706 18364699

[B59] Phadwal K. Kurian D. Salamat M. K. F. MacRae V. E. Diack A. B. Manson J. C. (2018). Spermine increases acetylation of tubulins and facilitates autophagic degradation of prion aggregates. *Sci. Rep.* 8 1–17. 10.1038/s41598-018-28296-y 29968775 PMC6030104

[B60] Pinho B. R. Reis S. D. Guedes-Dias P. Leitão-Rocha A. Quintas C. Valentão P. (2016). Pharmacological modulation of HDAC1 and HDAC6 in vivo in a zebrafish model: therapeutic implications for Parkinson’s disease. *Pharmacol. Res.* 103 328–339. 10.1016/j.phrs.2015.11.024 26657418

[B61] Pooranachandran N. Malicki J. J. (2016). Unexpected roles for ciliary kinesins and intraflagellar transport proteins. *Genetics* 203 771–785. 10.1534/genetics.115.180943 27038111 PMC4896193

[B62] Portran D. Schaedel L. Xu Z. Théry M. Nachury M. V. (2017). Tubulin acetylation protects long-lived microtubules against mechanical ageing. *Nat. Cell Biol.* 19 391–398. 10.1038/ncb3481 28250419 PMC5376231

[B63] Prodromou N. V. Thompson C. L. Osborn D. P. S. Cogger K. F. Ashworth R. Knight M. M. (2012). Heat shock induces rapid resorption of primary cilia. *J. Cell Sci.* 125 4297–4305. 10.1242/jcs.100545 22718348 PMC3516438

[B64] Pugacheva E. N. Jablonski S. A. Hartman T. R. Henske E. P. Golemis E. A. (2007). HEF1-Dependent Aurora A Activation Induces Disassembly of the Primary Cilium. *Cell* 129 1351–1363. 10.1016/j.cell.2007.04.035 17604723 PMC2504417

[B65] Ran J. Yang Y. Li D. Liu M. Zhou J. (2015). Deacetylation of α-tubulin and cortactin is required for HDAC6 to trigger ciliary disassembly. *Sci. Rep.* 5:12917. 10.1038/srep12917 26246421 PMC4526867

[B66] Reed N. A. Cai D. Blasius T. L. Jih G. T. Meyhofer E. Gaertig J. (2006). Microtubule Acetylation Promotes Kinesin-1 Binding and Transport. *Curr. Biol.* 16 2166–2172. 10.1016/j.cub.2006.09.014 17084703

[B67] Reiter J. F. Leroux M. R. (2017). Genes and molecular pathways underpinning ciliopathies. *Nat. Rev. Mol. Cell Biol.* 18 533–547. 10.1038/nrm.2017.60 PMC5851292 28698599

[B68] Renga V. (2019). Clinical Evaluation of Patients with Vestibular Dysfunction. *Neurol. Res. Int.* 2019:3931548. 10.1155/2019/3931548 30863640 PMC6377969

[B69] Riolo M. T. Cooper Z. A. Holloway M. P. Cheng Y. Bianchi C. Yakirevich E. (2012). Histone deacetylase 6 (HDAC6) deacetylates survivin for its nuclear export in breast cancer. *J. Biol. Chem.* 287 10885–10893. 10.1074/jbc.M111.308791 22334690 PMC3322878

[B70] Rocha C. Papon L. Cacheux W. Marques Sousa P. Lascano V. Tort O. (2014). Tubulin glycylases are required for primary cilia, control of cell proliferation and tumor development in colon. *EMBO J.* 33:2735 10.15252/embj.201490279 PMC4282510 25180231

[B71] Sander J. D. Joung J. K. (2014). Review CRISPR-Cas systems for editing, regulating and targeting genomes. *Nat. Biotechnol.* 32 347–355. 10.1038/nbt.2842 24584096 PMC4022601

[B72] Sheu J. R. Hsieh C. Y. Jayakumar T. Lin G. Y. Lee H. N. Huang S. W. (2019). HDAC6 dysfunction contributes to impaired maturation of adult neurogenesis in vivo: vital role on functional recovery after ischemic stroke. *J. Biomed. Sci.* 26 1–17. 10.1186/s12929-019-0521-1 30999900 PMC6471870

[B73] Shi Y. Su Y. Lipschutz J. H. Lobo G. P. (2017). Zebrafish as models to study ciliopathies of the eye and kidney. *Clin. Nephrol. Res.* 1 6–9. PMC5851006 29553143

[B74] Skoge R. H. Ziegler M. (2016). SIRT2 inactivation reveals a subset of hyperacetylated perinuclear microtubules inaccessible to HDAC6. *J. Cell Sci.* 129 2972–2982. 10.1242/jcs.187518 27311481

[B75] Song Y. Brady S. T. (2015). Post-translational modifications of tubulin: pathways to functional diversity of microtubules. *Trends Cell Biol.* 25 125–136. 10.1016/j.tcb.2014.10.004 25468068 PMC4344850

[B76] Stooke-Vaughan G. A. Huang P. Hammond K. L. Schier A. F. Whitfield T. T. (2012). The role of hair cells, cilia and ciliary motility in otolith formation in the zebrafish otic vesicle. *Development* 139 1777–1787. 10.1242/dev.079947 22461562 PMC3328178

[B77] Sullivan-Brown J. Schottenfeld J. Okabe N. Hostetter C. L. Serluca F. C. Thiberge S. Y. (2008). Zebrafish mutations affecting cilia motility share similar cystic phenotypes and suggest a mechanism of cyst formation that differs from pkd2 morphants. *Dev. Biol.* 314 261–275. 10.1016/j.ydbio.2007.11.025 18178183 PMC2453220

[B78] Szyk A. Deaconescu A. M. Spector J. Goodman B. Valenstein M. L. Ziolkowska N. E. (2014). Molecular basis for age-dependent microtubule acetylation by tubulin acetyltransferase. *Cell* 157 1405–1415. 10.1016/j.cell.2014.03.061 24906155 PMC4726456

[B79] Tang X. Gao J. S. Guan Y-jie McLane K. E. Yuan Z. L. Ramratnam B. (2007). Acetylation-Dependent Signal Transduction for Type I Interferon Receptor. *Cell* 131 93–105. 10.1016/j.cell.2007.07.034 17923090

[B80] Tong J. J. Liu J. Bertos N. R. Yang X.-J. (2002). Identification of HDAC10, a novel class II human histone deacetylase containing a leucine-rich domain. *Nucleic Acids Res.* 30 1114–1123. 10.1093/nar/30.5.1114 11861901 PMC101247

[B81] Vaquero A. Scher M. B. Dong H. L. Sutton A. Cheng H. L. Alt F. W. (2006). SirT2 is a histone deacetylase with preference for histone H4 Lys 16 during mitosis. *Genes Dev.* 20 1256–1261. 10.1101/gad.1412706 16648462 PMC1472900

[B82] Viau A. Bienaimé F. Lukas K. Todkar A. P. Knoll M. Yakulov T. A. (2018). Cilia-localized LKB 1 regulates chemokine signaling, macrophage recruitment, and tissue homeostasis in the kidney. *EMBO J.* 37 1–21. 10.15252/embj.201798615 29925518 PMC6068446

[B83] Waters A. M. Beales P. L. (2011). Ciliopathies: an expanding disease spectrum. *Pediatr. Nephrol.* 26 1039–1056. 10.1007/s00467-010-1731-7 21210154 PMC3098370

[B84] Whatley M. Francis A. Ng Z. Y. Khoh X. E. Atlas M. D. Dilley R. J. (2020). Usher Syndrome: genetics and Molecular Links of Hearing Loss and Directions for Therapy. *Front. Genet.* 11:565216 10.3389/fgene.2020.565216 33193648 PMC7642844

[B85] Whitfield T. T. (2020). Cilia in the developing zebrafish ear. *Philos. Trans. R. Soc. Lond. B Biol. Sci.* 375:20190163. 10.1098/rstb.2019.0163 31884918 PMC7017339

[B86] Wloga D. Webster D. M. Rogowski K. Bré M. H. Levilliers N. Jerka-Dziadosz M. (2009). TTLL3 Is a tubulin glycine ligase that regulates the assembly of cilia. *Dev. Cell* 16, 867–876. 10.1016/j.devcel.2009.04.008 19531357

[B87] Yamaguchi M. (2005). Histone deacetylase 1 regulates retinal neurogenesis in zebrafish by suppressing Wnt and Notch signaling pathways. *Development* 132 3027–3043. 10.1242/dev.01881 15944187

[B88] Yu D. S. Song X. L. Yan C. (2021). Oncogenic miRNA-1908 targets HDAC10 and promotes the aggressive phenotype of cervical cancer cell. *Kaohsiung J. Med. Sci.* 1–9. 10.1002/kjm2.12348 33493381 PMC11896177

[B89] Zhang X. Yuan Z. Zhang Y. Yong S. Salas-Burgos A. Koomen J. (2007). HDAC6 Modulates Cell Motility by Altering the Acetylation Level of Cortactin. *Mol. Cell* 27 197–213. 10.1016/j.molcel.2007.05.033 17643370 PMC2684874

[B90] Zhang Y. Kwon S. Yamaguchi T. Cubizolles F. Rousseaux S. Kneissel M. (2008). Mice Lacking Histone Deacetylase 6 Have Hyperacetylated Tubulin but Are Viable and Develop Normally. *Mol. Cell. Biol.* 28 1688–1701. 10.1128/MCB.01154-06 18180281 PMC2258784

[B91] Zhang Y. Ying J. B. Hong J. J. Li F. C. Fu T. T. Yang F. Y. (2019). How does chirality determine the selective inhibition of histone deacetylase 6? a lesson from trichostatin a enantiomers based on molecular dynamics. *ACS Chem. Neurosci.* 10, 2467–2480. 10.1021/acschemneuro.8b00729 30784262

[B92] Zhao Y. Yang J. Liao W. Liu X. Zhang H. Wang S. (2010). Cytosolic FoxO1 is essential for the induction of autophagy and tumour suppressor activity. *Nat. Cell Biol.* 12 665–675. 10.1038/ncb2069 20543840

[B93] Zhou X. Fan L. X. Li K. Ramchandran R. Calvet J. P. Li X. (2014). SIRT2 regulates ciliogenesis and contributes to abnormal centrosome amplification caused by loss of polycystin-1. *Hum. Mol. Genet.* 23 1644–1655. 10.1093/hmg/ddt556 24203696 PMC3929098

[B94] Zu Y. Tong X. Wang Z. Liu D. Pan R. Li Z. (2013). TALEN-mediated precise genome modification by homologous recombination in zebrafish. *Nat. Methods* 10 329–331. 10.1038/nmeth.2374 23435258

