# Supplementary figures and images for: Loss of Deacetylation Enzymes Hdac6 and Sirt2 Promotes Acetylation of Cytoplasmic Tubulin, but Suppresses Axonemal Acetylation in Zebrafish Cilia
Source: Front Cell Dev Biol. 2021 Jun 28;9:676214. doi: 10.3389/fcell.2021.676214 (PMC8276265; doi:10.3389/fcell.2021.676214)

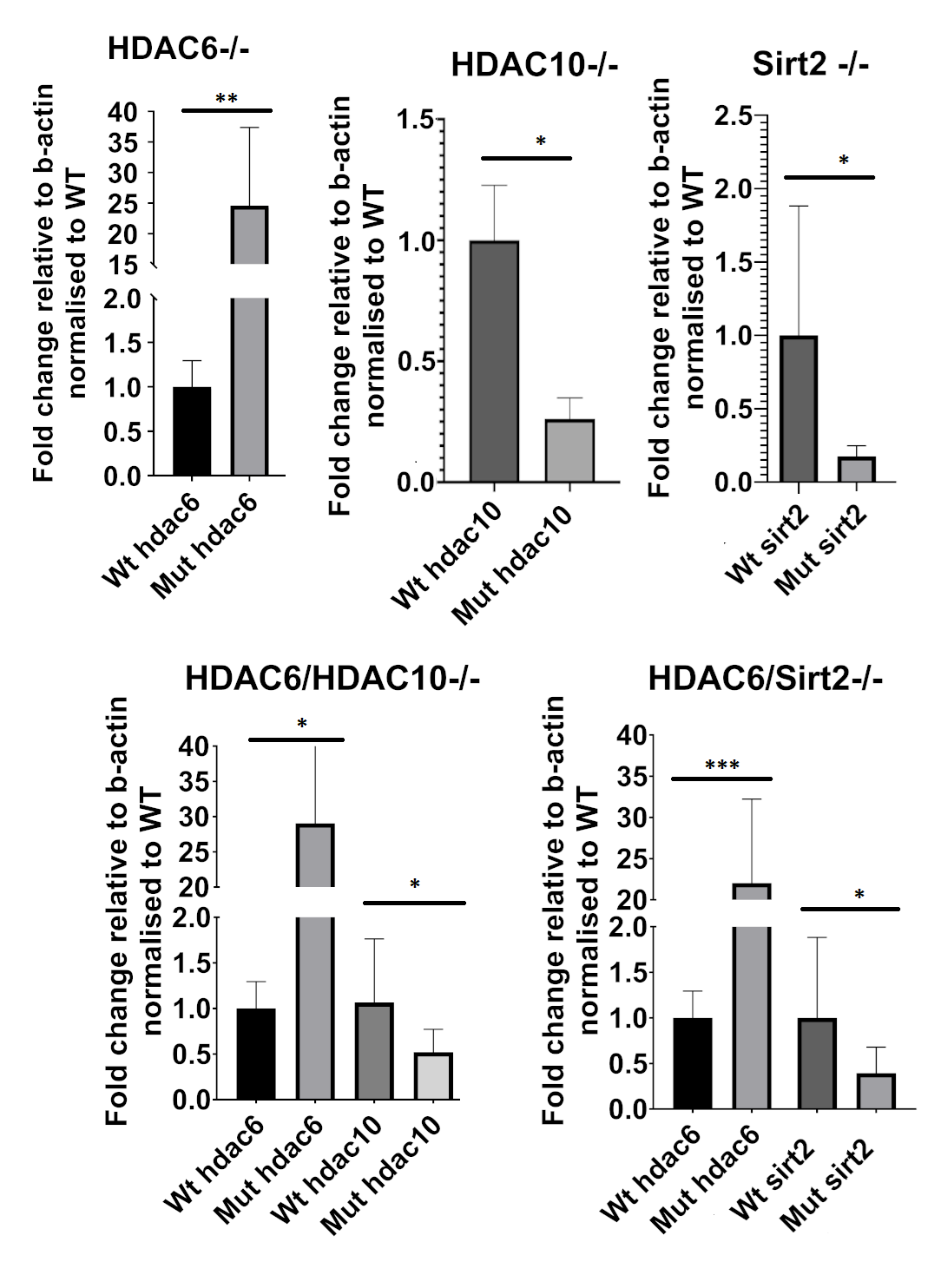

Supplement: Supplementary Figure 1 — Levels of mRNA expression for all three genes in various single and double mutants. The mutant is indicated above the panel the gene that was analyzed for expression is indicated on the X-axis. Wt: pooled wild-type siblings. Mut: pooled mutants (A–C) or double mutants (D,E) (A) hdac6-/- B) hdac10-/- (C) sirt2-/- single mutants and (D) hdac6-/-/hdac10-/- and hdac6-/-/sirt2-/- double mutants. Values normalized to actin expression level and then to wild type values. (Mean with SEM for n = 3. p < 0.05). [file Image_1.TIF]

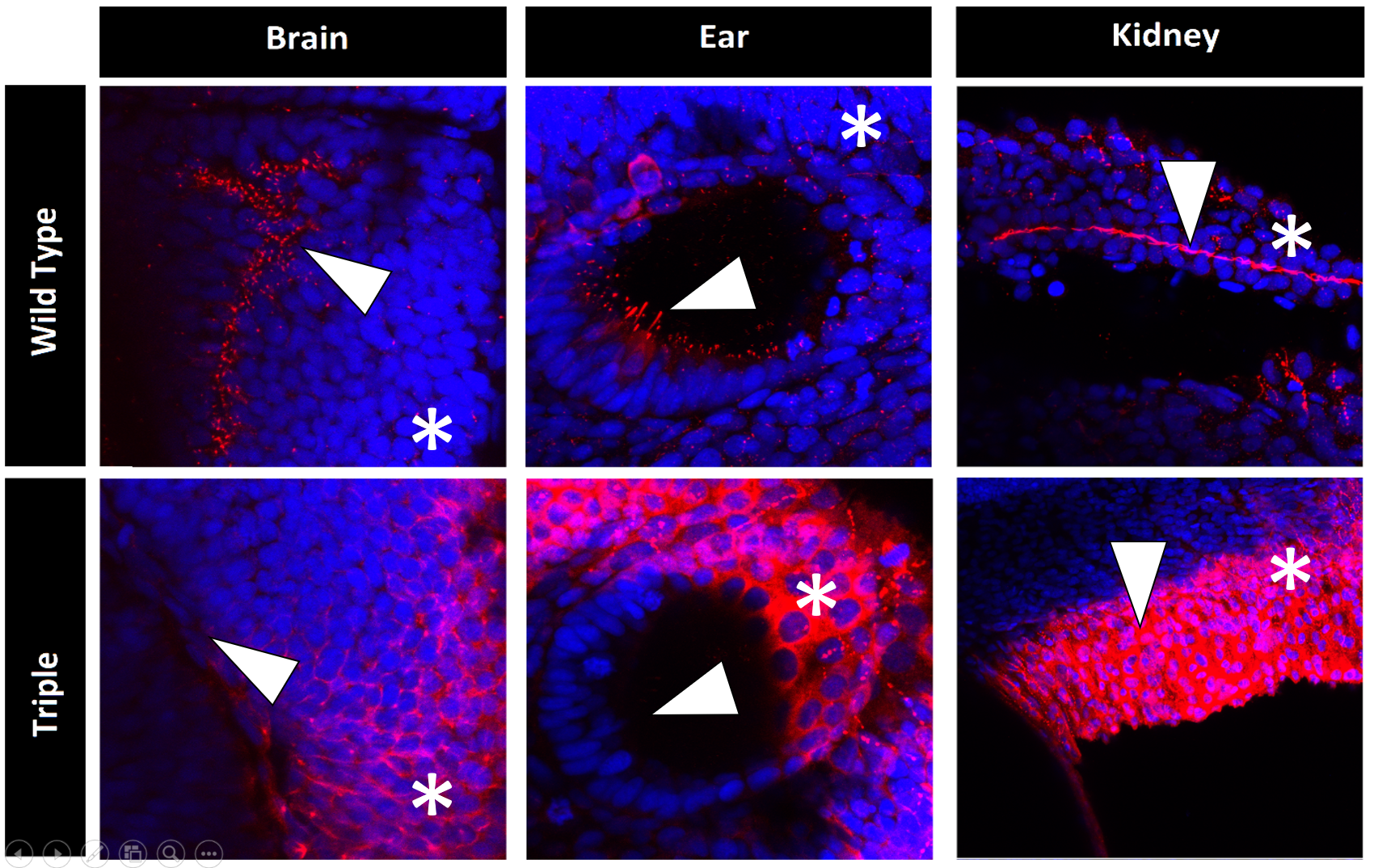

Supplement: Supplementary Figure 2 — Hyperacetylation of tubulin in brain cells surrounding ear and kidney and hypoacetylation in cilia at 1 dpf. Red Acetyl-α-Tubulin (Lys40) (6-11B-1) Mouse mAb blue DAPI. Asterisks show hyperacetylated tubulin within cell bodies. Arrowheads shows cilia positions. [file Image_2.TIF]

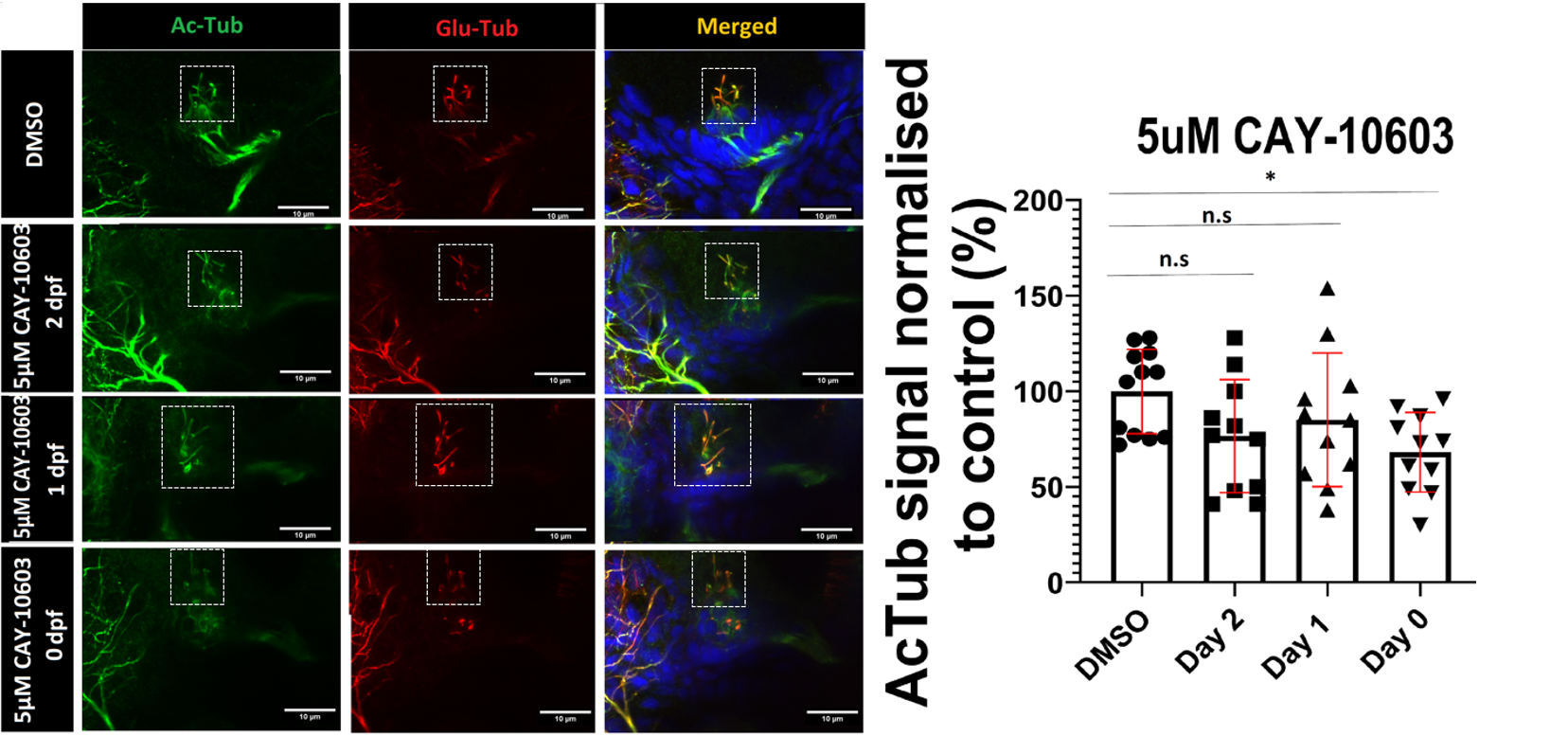

Supplement: Supplementary Figure 3 — Early inhibition (6 hpf) of hdac6 is required to trigger hypoacetylation phenotype in cilia in cristae at 3 dpf. Acetylated tubulin green glutamylated red DAPI blue. White boxes show cilia position. Mean with CI95% nwt = 12 treated = 11. p < 0.05. [file Image_3.TIF]

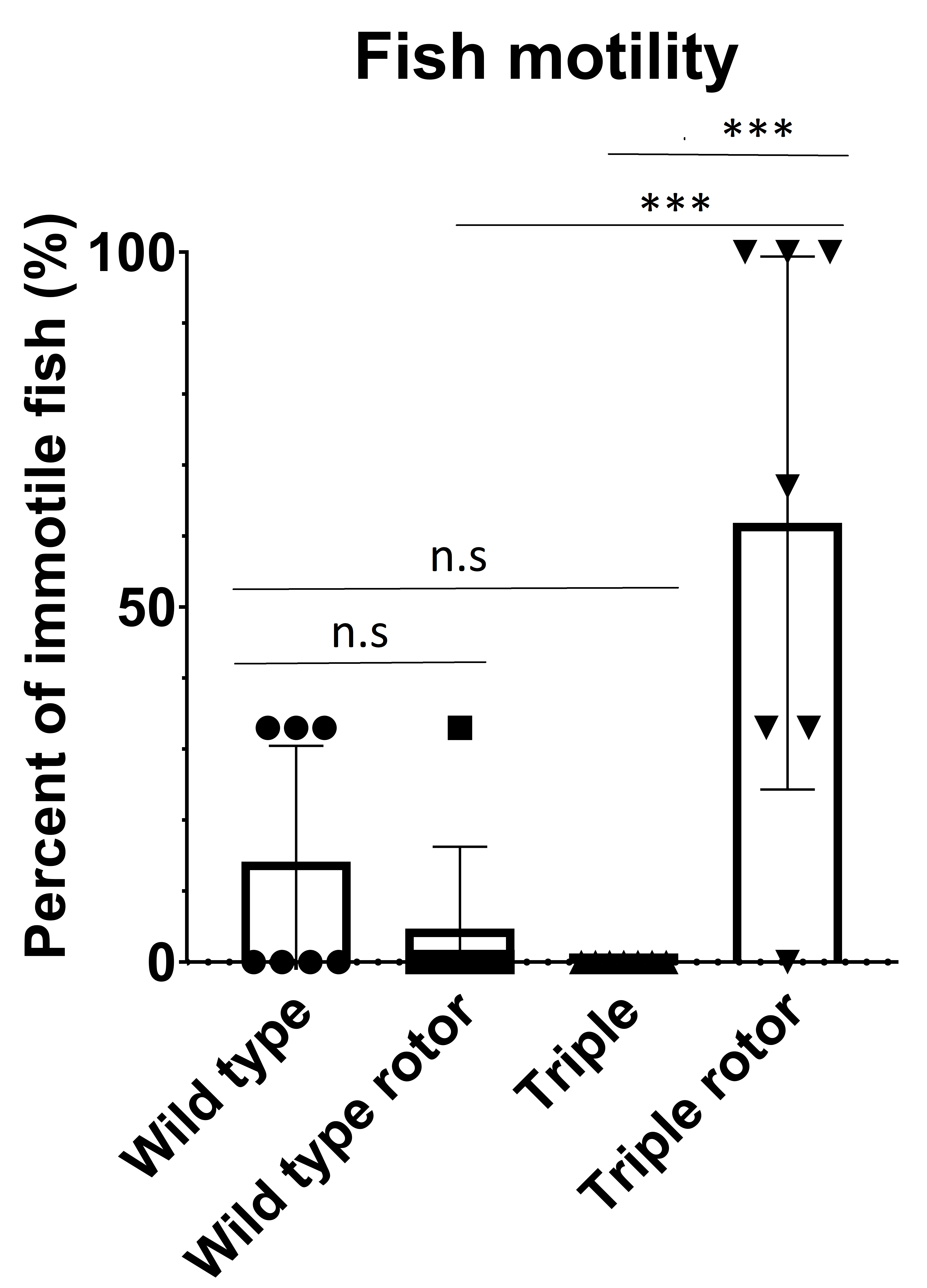

Supplement: Supplementary Figure 4 — Immotile fish after rotor treatment. Percent of fish which swam less than 20% of distance, swam by average of untreated wild type (three larvae per trail). Mean with CI-95% One-way ANOVA p < 0.05. [file Image_4.TIF]

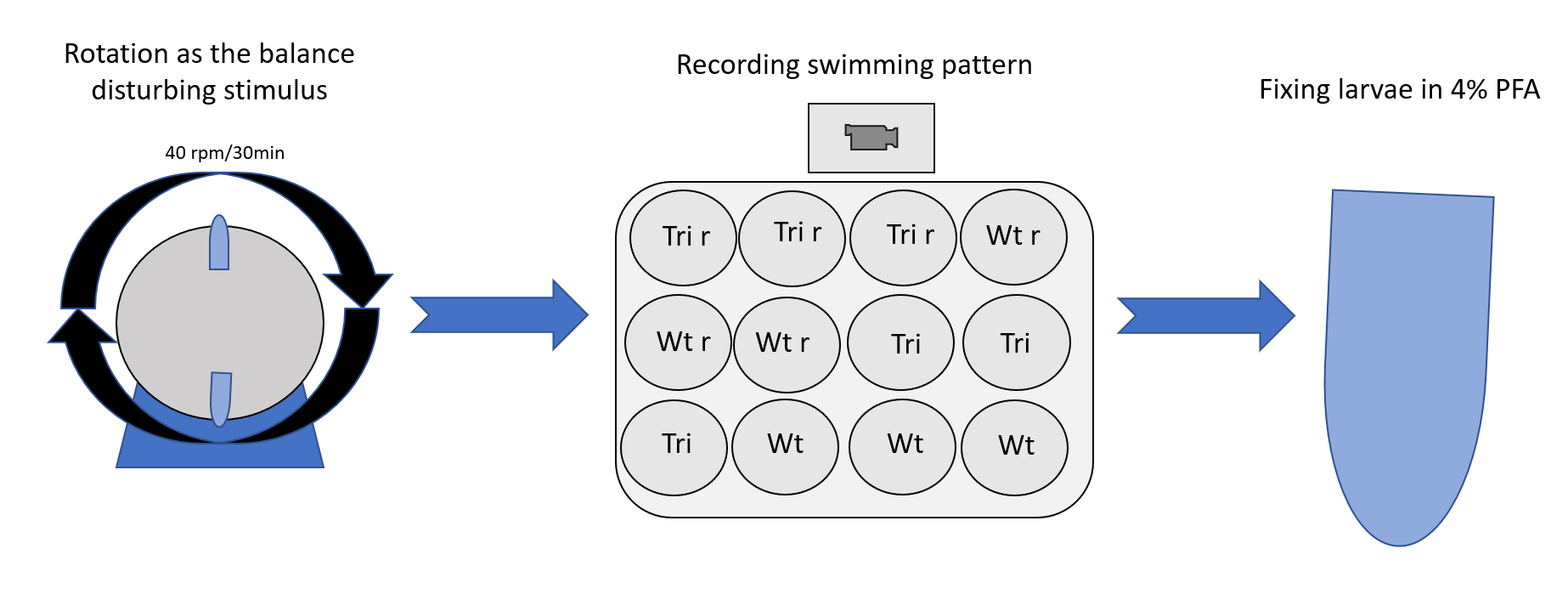

Supplement: Supplementary Figure 5 — Schematic representation of disturbing balance experiment in zebrafish larvae. Wt, Wild type; Wt r, Wild type after treatment; Tri, Triple mutant; Tri r, Triple mutant after treatment. [file Image_5.TIF]

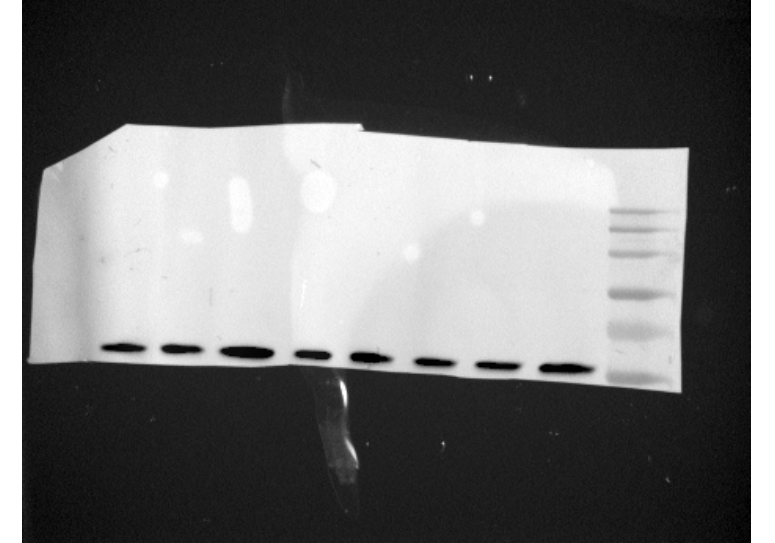

Supplement: Supplementary file 6 [file Image_6.TIF]

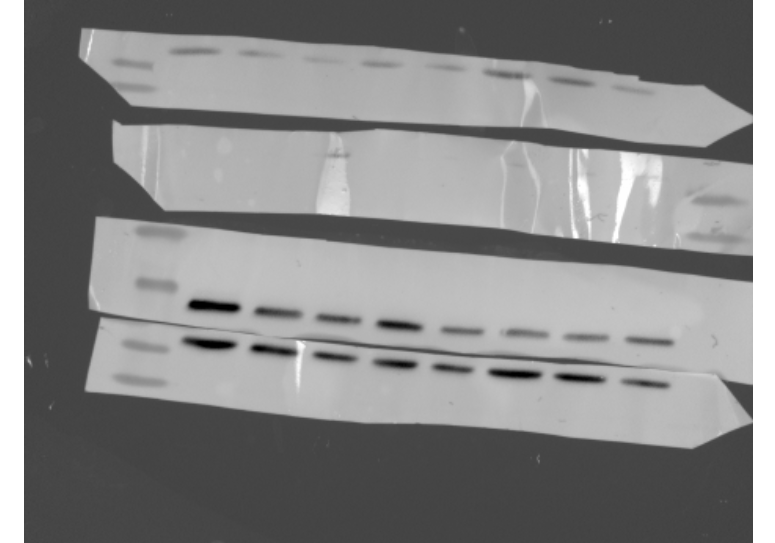

Supplement: Supplementary file 7 [file Image_7.TIF]

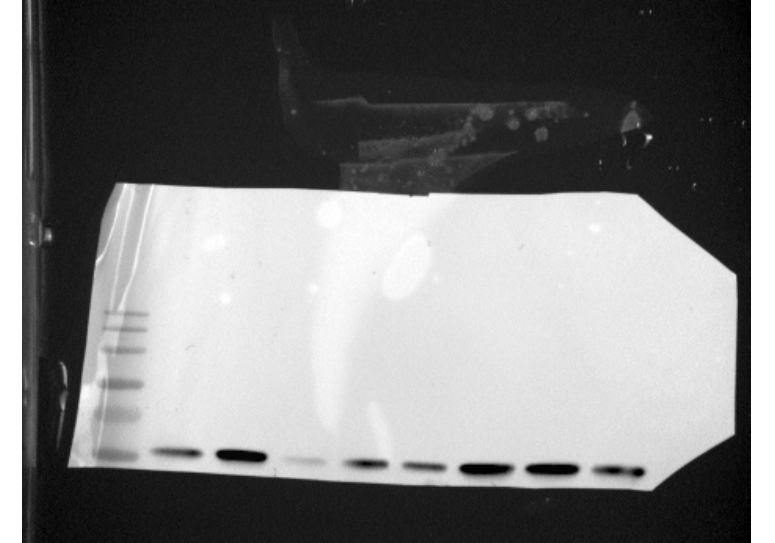

Supplement: Supplementary file 8 [file Image_8.TIF]
